# The impact of cardiovascular events in bronchiectasis: a systematic review and meta-analysis

**DOI:** 10.1183/23120541.01032-2023

**Published:** 2024-09-30

**Authors:** Andrea Gramegna, Ivan Barone, Gianfranco Alicandro, Giovanni Sotgiu, Angela Bellofiore, Crizia Colombo, Antonella Arcadu, Margherita Ori, Federico Blasi, Edoardo Simonetta, Marco Vicenzi, Stefano Aliberti, Francesco Blasi

**Affiliations:** 1 Department of Pathophysiology and Transplantation, University of Milan, Milan, Italy; 2 Respiratory Unit and Cystic Fibrosis Adult Center, Fondazione IRCCS Ca’ Granda Ospedale Maggiore Policlinico, Milan, Italy; 3 Department of Paediatrics, Cystic Fibrosis Center, Fondazione IRCCS Ca’ Granda Ospedale Maggiore Policlinico, Milan, Italy; 4 Department of Medical, Surgical and Experimental Sciences, Clinical Epidemiology and Medical Statistics Unit, University of Sassari, Sassari, Italy; 5 Healthcare Professions Department, Fondazione IRCCS Ca’ Granda Ospedale Maggiore Policlinico, Milan, Italy; 6 Department of Cardio-Thoracic-Vascular Area, Cardiology Unit, Foundation IRCCS Ca’ Granda Ospedale Maggiore Policlinico, Milan, Italy; 7 Department of Medicine and Surgery, University of Insubria, Varese, Italy; 8 Cardiology Division, ASST Rhodense, Rho, Italy; 9 Respiratory Unit, IRCCS Humanitas Research Hospital, Rozzano, Italy; 10 Dyspnea Lab, Department of Clinical Sciences and Community Health, University of Milan, Milan, Italy; 11 Department of Biomedical Sciences, Humanitas University, Milan, Italy

## Abstract

**Background:**

Bronchiectasis is a chronic respiratory condition characterised by airway and systemic inflammation with prevalence increasing with age. Given the median age of the patients, it is common to observe the presence of comorbidities, particularly cardiovascular diseases, which have been linked to adverse clinical outcomes. To investigate the pooled estimates of the association between bronchiectasis and coronary heart disease or stroke within this population, we conducted a systematic review and meta-analysis of the available scientific evidence.

**Methods:**

Three investigators independently performed the search on PubMed and other sources and included studies published up to October 2023 according to predefined criteria. Relative measures of association between bronchiectasis and cardiovascular events were pooled and meta-analysed using a fixed-effects model. Studies were evaluated using the Newcastle-Ottawa Scale for assessing the quality of non-randomised studies in meta-analyses.

**Results:**

A final pool of nine studies was included in the systematic review, with a total of 22 239 patients. Meta-analysis of three high-quality cohort studies showed a pooled hazard ratio of 1.42 (95% CI 1.30–1.57) for coronary heart disease and 1.71 (95% CI 1.55–1.89) for cerebrovascular stroke.

**Conclusions:**

The increased cardiovascular risk among people with bronchiectasis underscores the critical need to raise awareness of this association and to develop preventive strategies accordingly. Further translational studies are imperative to gain a deeper understanding of the complex interplay between inflammation, the immune system and endothelial dysfunction in this patient group.

## Shareable abstract

**People with bronchiectasis have an increased risk for cardiovascular events, including coronary heart disease and cerebrovascular stroke** https://bit.ly/4bgFvI1

## Introduction

Bronchiectasis is a chronic respiratory condition characterised by abnormal and persistent dilation of the bronchi, accompanied by symptoms such as chronic cough, sputum production and a history of exacerbations [1]. Recent epidemiological studies have revealed an average prevalence ranging from 701 to 1200 per 100 000 population, with rates increasing with age [2–5]. The mean age of bronchiectasis patients typically falls between 60 and 70 years in European and American studies [6].

Given the median age of these patients, comorbidities are common and have been linked to worse clinical outcomes, including exacerbation frequency, lung function decline, diminished quality of life and increased mortality [7–10]. Cardiovascular (CV) diseases are among the most prevalent comorbidities in bronchiectasis patients, with factors associated with CV events, such as aortic stiffness and cardiac biomarkers, worsening as the disease progresses [11].

The increased CV risk in bronchiectasis patients has been attributed, in part, to advancing age. While the precise mechanisms underlying CV manifestations remain unclear, this association may also be attributable to the substantial burden of chronic systemic inflammation and oxidative stress observed in this population, as seen in other chronic conditions [11–14].

Some studies have reported a higher incidence of myocardial infarction and cerebrovascular stroke following severe pulmonary exacerbations or other lower respiratory tract infections, indicating increased mortality from CV events [15–20]. However, much of the evidence on CV risk is derived from a limited number of retrospective studies, and no study to date has systematically investigated the association between bronchiectasis and the risk of CV events [21]. A deeper understanding of this relationship could have significant implications for clinical monitoring and preventive strategies in this patient population.

Therefore, this systematic review aims to explore the association between bronchiectasis and coronary heart disease (CHD) or stroke within this population. We systematically collected and summarised observational cohort studies reporting the incidence and characteristics of CV events in bronchiectasis patients, conducting a systematic review and meta-analysis of the available scientific evidence.

## Methods

### Search methodology

After registration of the review protocol in the international prospective register PROSPERO (ID CRD42022330081), three investigators (AA, CC and IB) independently conducted an electronic search on PubMed and other databases (Cochrane Library, Embase, Ovid). Studies published from 1 January 2000 to 1 October 2023 were considered. The search string used for the electronic search was: (“bronchiectasis”[All Fields] OR “bronchiectasis”[MeSH Terms] OR “bronchiectasis”[All Fields] OR “bronchiectasis”[All Fields]) AND (“cardiovascular diseases”[MeSH Terms] OR (“cardiovascular”[All Fields] AND “diseases”[All Fields]) OR “cardiovascular diseases”[All Fields] OR (“cardiovascular”[All Fields] AND “disease”[All Fields]) OR “cardiovascular disease”[All Fields]). This systematic review was conducted according to the Preferred Reporting Items for Systematic Meta-Analyses (PRISMA) statement [22].

### Study selection

Titles and abstracts were screened by two independent investigators (AG and IB). We also manually searched the references included in the full text of the eligible articles to identify any studies that might have been missed. In case of disagreement a final decision was taken by the principal investigator (AG). Records were excluded if 1) they were written in languages other than English, 2) they were case reports, case series or qualitative reviews, 3) they included paediatric patients (<18 years of age), 4) they included patients affected by cystic fibrosis or other genetically determined causes for bronchiectasis (*e.g.* primary ciliary dyskinesia) or 5) the full text was unavailable. Non-peer-reviewed papers were not selected owing to poor methodological reliability. The full text was then obtained for selected papers.

### Data extraction

Following a comprehensive analysis of the full texts, relevant information was extracted from each paper. The extracted data included the year of publication, study design, number of included patients, type of controls (if any), participant age, bronchiectasis severity, study outcomes and main results. We searched each paper for any measure of association among odds ratio (OR), relative risk and hazard ratio (HR) and their corresponding 95% confidence intervals (CIs). Additionally, covariates employed for adjustments and collected risk factors associated with CV events were also collected.

### Quantitative synthesis of the evidence

When at least two different studies provided a relative measure of association between bronchiectasis and CV events, the data were pooled using a meta-analytic approach. Specifically, a fixed-effects model was used to obtain a pooled estimate of the relative risk for the outcomes considered in the retrieved studies [23]. The pooled estimate was calculated as a weighted average of study-specific relative risk, for which the weights assigned to each study were the inverse of the study's variance.

### Critical assessment of evidence quality

Studies included in the quantitative synthesis were evaluated using the Newcastle-Ottawa Scale for assessing the quality of non-randomised studies in meta-analyses (www.ohri.ca/programs/clinical_epidemiology/oxford.asp). This scale is based on a “star system” in which a study is judged on three perspectives: the selection of the study groups, the comparability of the groups and the ascertainment of the outcome of interest. Selection of the study groups can receive a maximum of four stars, comparability of the groups up to two stars and ascertainment of the study outcome up to three stars.

## Results

### Summary of evidence

**FIGURE 1:**
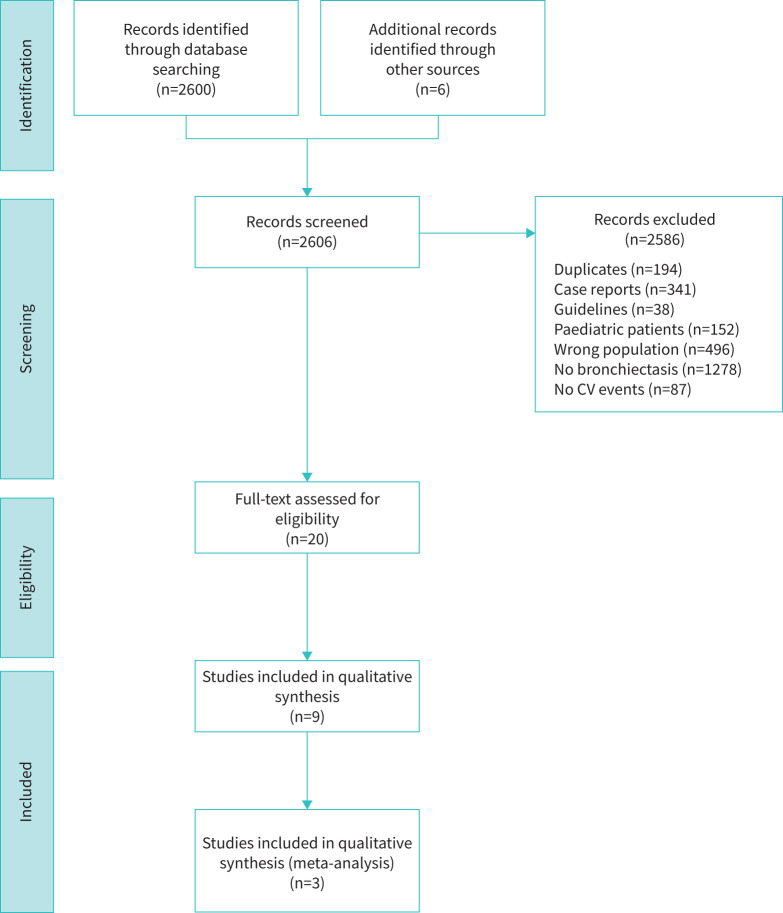
Flow chart of study selection. CV: cardiovascular.

The electronic search initially identified 2600 records. The majority of the selected studies were excluded because they did not involve bronchiectasis patients (n=1278), focused on cystic fibrosis or other genetically determined causes of bronchiectasis (n=496) or involved paediatric patients (n=152). Additionally, 87 papers were excluded for not evaluating CV events. We also excluded 341 case reports and case series, along with 38 guidelines or recommendations. Six additional papers were identified through manual assessment of the references cited in the selected papers. After eliminating 194 duplicates, 20 papers were subjected to full-text analysis (figure 1). A final pool of nine papers were included in the qualitative analysis, with a total of 22 239 bronchiectasis patients. The selected studies were published between 2016 and 2022 (table 1).

**TABLE 1:** Articles retrieved by the electronic search

Study	Study design	Population	Controls	Median age	Median follow-up	Outcome	Results	Study quality assessment^#^
**Navaratnam *et al.* 2017 [24]**	Retrospective cohort	10 942 patients included in the electronic primary care data from the CPRD (UK)	3 884 770 individuals included in the CPRD without BE	47 years	5.6 years	Cross-sectional analysis: existing diagnoses of CHD^¶^ and stroke^+^ prior to the index date Historical cohort analysis: first-time diagnoses of CHD and stroke after the index date were considered as incident events	Cross-sectional analysis: BE was associated with increased odds for CHD (OR 1.33, 95% CI 1.25–1.41), stroke (OR 1.92, 95% CI 1.85–2.01), angina (OR 1.33, 95% CI 1.24–1.43), CABG (OR 1.87, 95% CI 1.65–2.17) and MI (OR 1.11, 95% CI 1.01–1.22) Historical cohort analysis: crude rates of first CHD event in people with and without BE were 6.6 per 1000 person-years (95% CI 5.9–7.5 per 1000 person-years) and 2.2 per 1000 person-years, respectively The adjusted HR was 1.44 (95% CI 1.27–1.63) The estimate was adjusted for sex, age, smoking, diabetes, hypertension, hyperlipidaemia and family history of CVD	Selection: ★★★★ Comparability: ★★ Outcome: ★★★
**Chen *et al.* 2017 [25]**	Retrospective cohort	1295 patients retrieved from the NHI Research Database (Taiwan)	6475 individuals, frequency-matched by age and sex, selected from the general population without BE	62 years	4.9 and 5.4 years for patients with BE and controls, respectively^¶^	Incidence and risk of ischaemic stroke	Higher risk of ischaemic stroke in patients with BE as compared to controls (HR 1.74, 95% CI 1.28–2.35) The estimate was adjusted for age, sex and comorbidities	Selection: ★★★★ Comparability: ★★ Outcome: ★★★
**Evans *et al.* 2017 [26]**	Retrospective cohort	400 patients attending the BE service Edinburgh (UK)	None	66 years	Not reported	Prevalence of CVD and risk factors	45 patients (11.3%) developed vascular disease after the diagnosis of BE BSI was associated with increased odds of CVD (OR for scores 5–8: 3.92, 95% CI 1.21–12.71; OR for scores ≥9: 8.12, 95% CI 2.44–27.0)	Selection: ★★☆☆ Comparability: ☆☆ Outcome: ★★★
**Menéndez *et al.* 2017 [27]**	Prospective cohort	265 patients attending the specialised outpatient clinics of two tertiary care university hospitals, Spain	None	68.4 years	1 year	To evaluate factors associated with exacerbations requiring hospital admission	HF at baseline was associated with exacerbation requiring hospital admission (OR 5.47, 95% CI 1.36–37.23), while MI was not (OR 0.72, 95% CI 0.13–6.06) The estimates were adjusted for FACED score Similar estimates were obtained when adjusting for BSI	Selection: ★★★★ Comparability: ☆☆ Outcome: ☆☆☆
**Navaratnam *et al.* 2017 [24]**	Retrospective cohort	895 patients selected from a larger cohort of 26 518 individuals included in the electronic primary care data from the CPRD, who had both a first CV event and at least one RTI during the study observation period (UK)	Self-controlled cases	Age categories: <45 years: 16.3% 45–55 years: 12.7% 56–65 years: 20.9% 66–75 years: 27.7% >75 years: 22.4%	Incidence rate ratios of a first CV event evaluated at different time points up to 91 days after RTI	First record of a CV event, a composite outcome of first recorded diagnosis of MI or stroke	Compared to a baseline period (before RTI) the incidence rate ratios were: 2.39 (95% CI 1.21–5.62) during the first 3 days post RTI, 2.01 (1.22–2.78) 4–7 days after, 1.73 (1.09–2.13) 8–14 days after, 1.16 (0.77–2.19) 15–28 days after and 1.08 (0.69–1.53) 29–91 days after The estimates were adjusted for age and season	Selection: ★★★★ Comparability: ★★ Outcome: ★★★
**Hung *et al.* 2018 [28]**	Retrospective cohort	7156 patients included in the Longitudinal Health Insurance Database 2000, a national database comprising data of 1 million randomly selected beneficiaries of the NHI programme in 2000 (Taiwan)	14 084 individuals without BE, selected from the general population and frequency-matched according to sex, age and entry year	63.3 years	2.4 person-years for patients with BE and 5.2 person-years among controls^§^	The primary outcome was an ACS event	ACS incidence was higher in the BE cohort than in the comparison cohort (13.49 *versus* 9.07 per 1000 person-years) Adjusted HR 1.40 (95% CI 1.20–1.61) The estimate was adjusted for age, sex and comorbidities	Selection: ★★★★ Comparability: ★★ Outcome: ★★★
**Chen *et al.* 2020 [29]**	Retrospective cohort	603 inpatients diagnosed with BE in the Affiliated Yancheng Hospital of Southeast University Medical College (Jiangsu, China)	None	62 years among patients without CV comorbidities and 65.4 years among patients with CV comorbidities	Prevalence of CV comorbidities was evaluated only at baseline	CV comorbidity was defined as a composite outcome of having a history of CHD (ACS, chronic coronary artery disease), cerebrovascular events (including ischaemic stroke, haemorrhagic stroke or TIA), PAD or HF	199 patients (33.0%) had a history of CV event Main CV event registered: ACS: 81 (13.4%), CAD: 23 (3.8%), ischaemic stroke: 37 (6.1%), haemorrhagic stroke: 8 (1.3%), TIA: 58 (9.6%), PAD: 24 (4.0%), HF: 29 (4.8%)	Selection: ★★☆☆ Comparability: ☆☆ Outcome: ★☆☆
**Huang *et al.* 2020 [21]**	Longitudinal cohort	433 patients	None	67 years	61.4 months per participant	All-cause and CV mortality	Increasing serum desmosine concentrations were associated with increasing all-cause mortality (HR 2.30, 95% CI 1.85–2.84; p<0.0001) Serum desmosine was associated with increased cardiovascular mortality (HR 2.21, sd 95% CI 1.60–3.05; p<0.0001)	Selection: ★★★ Comparability: ☆☆ Outcome: ★☆☆
**Méndez *et al.* 2022 [30]**	*Post hoc* retrospective analysis of a prospective observational study	250 patients enrolled at two tertiary care hospitals (Spain)	None	72 years	35 months	CV events were defined as any ACS, new or worsening HF, new or recurrent arrhythmia requiring hospital admission or emergency department care, or cerebrovascular accident (stroke or TIA)	74 patients (29.6%) had a CV event	Selection: ★★ Comparability: ☆☆ Outcome: ★★★

ACS: acute coronary syndrome (defined as acute MI or unstable angina); BE: bronchiectasis; CAD: coronary artery disease; CHD: coronary heart disease; CI: confidence interval; CABG: coronary artery bypass graft; CPRD: Clinical Practice Research Datalink; CVD: cardiovascular disease; CV: cardiovascular; HF: heart failure; MI: myocardial infarction; NHI: National Health Insurance; OR: odds ratio; PAD: peripheral artery disease; RTI: respiratory tract infection; TIA: transient ischaemic attack. ^#^: studies were evaluated used the Newcastle-Ottawa Scale, which is based on a “star system” in which a study is judged on three perspectives: the selection of the study groups (maximum of four stars), the comparability of the groups (maximum of two stars) and the ascertainment of the outcome of interest (maximum of three stars). Each star awarded to a study is represented by a filled star, and the total of filled and unfilled stars indicates the maximum stars attainable in each domain; ^¶^: CHD was a composite outcome of having at least one recorded diagnosis of angina (including unstable angina), MI or CABG; ^+^: stroke included ischaemic or haemorrhagic stroke, transient ischaemic attack and subarachnoid haemorrhage; ^§^: computed from table 2 of the original manuscript by dividing the person-years by the number of patients.

Chen *et al.* [25] in 2017 conducted a prospective study on 1295 bronchiectasis patients enrolled between 2000 and 2008 and 6475 controls without bronchiectasis among 1 million randomly selected beneficiaries of the Taiwanese National Health Insurance Database. Subjects were followed up to the date of ischaemic stroke, censoring or the end of 2010. During follow-up, 163 ischaemic strokes were observed among bronchiectasis patients and 58 among controls, with a rate of 9.1 per 1000 person-years among bronchiectasis patients and 4.7 among controls. After adjusting for sex, age and comorbidities (hypertension, diabetes, hyperlipidaemia, chronic artery disease, myocardial infarction, ischaemic heart disease, angina pectoris, chronic heart failure, COPD and atrial fibrillation), bronchiectasis patients had a 74% excess risk of ischaemic stroke (HR 1.74, 95% CI 1.28–2.35). The excess risk was even higher among patients with at least one comorbidity at baseline (HR 2.66, 95% CI 1.85–3.84).

The retrospective study by Evans *et al*. [26] conducted in 2017 included 400 patients from a National Health Service Lothian bronchiectasis clinic in Edinburgh (UK) between May 2013 and September 2014. Their aim was to determine the prevalence of vascular diseases (such as ischaemic heart disease, cerebrovascular disease, peripheral vascular disease and atrial fibrillation) in bronchiectasis patients. Data from the study showed that 11% of patients had a diagnosis of vascular disease prior to bronchiectasis diagnosis, while an additional 11% developed CV disease in the following period, with an average onset occurring after 9.4 years. Independent risk factors for CV disease in bronchiectasis patients were male sex, arterial hypertension, long-term statin therapy and moderate-to-severe bronchiectasis, suggesting that the severity of bronchiectasis is independently linked to the onset of vascular disease.

The prospective study from Menéndez *et al.* [27] enrolled individuals from two tertiary care university hospitals in Spain during the period 2011–2015. Pulmonary exacerbations requiring the administration of antibiotics were tracked over a year. The study included 265 patients with 162 hospitalisations during the follow-up. Independent risk factors for hospital admission were age, prior bronchiectasis-related hospitalisations, proton pump inhibitor use, heart failure and disease severity according to validated scores (Bronchiectasis Severity Index (BSI) and FACED).

In 2017, Navaratnam *et al.* [24] published a cross-sectional study based on registry data. The authors examined CV risk factors and medication usage in individuals with and without bronchiectasis. A number of the 10 942 participants had bronchiectasis. This analysis showed differences in CV risk factors and medication use between bronchiectasis patients and the general population. The lower prevalence of chronic CV diseases suggested that other factors related to bronchiectasis may play a role in increasing the risk of CV events in this population.

Navaratnam *et al.* [24] also analysed primary care electronic records from the Clinical Practice Research Datalink to conduct a cross-sectional study employing logistic regression to assess the association between bronchiectasis and CV events, defined as CHD or stroke. The findings revealed that bronchiectasis patients showed high prevalence of pre-existing CHD (OR 1.33, 95% CI 1.25–1.41) and stroke (OR 1.92, 95% CI 1.85–2.01) compared to those without bronchiectasis, even after adjusting for age, sex, smoking and other risk factors for CV disease. In addition, the incidence rate of CHD and stroke events was higher in bronchiectasis patients than in the comparison group (HR for CHD 1.44, 95% CI 1.27–1.63; HR for stroke 1.71, 95% CI 1.54–1.90).

In a case–control study from 2018, Hung *et al.* [28] enrolled 3521 bronchiectasis patients and matched with 14 084 general population participants based on sex, age and index year. Groups were followed from 2000 until the end of 2010 to assess the incidence of acute coronary syndrome (ACS). Data from the study highlighted that the overall risk of ACS was 40% higher in the bronchiectasis cohort (adjusted HR 1.40, 95% CI 1.20–1.62). The ACS risk was significantly elevated for those bronchiectasis patients with a higher incidence of respiratory infection-related emergency room visits or hospitalisations, showing a 5.46-fold and 8.15-fold increase, respectively.

The 2020 study from Chen *et al.* [29] retrospectively examined 603 consecutive inpatients with bronchiectasis at a hospital in China from 2014 to 2017. During their initial hospitalisation, symptoms, bacterial cultures, blood indicators and chest computed tomography scans were evaluated. During this period, 335 patients experienced at least one bronchiectasis exacerbation. The study identified independent risk factors for pulmonary exacerbations, including the presence of CV diseases, isolation of *Pseudomonas aeruginosa* and extension of radiological involvement.

Despite advancements in research on the topic, the risk factors that lead to an increased CV risk in patients with bronchiectasis are still largely unexplored. Some biomarkers have been studied to try and stratify patients with bronchiectasis based on CV risk. The longitudinal cohort study from Huang *et al.* [21] from 2020 found that serum desmosine predicted future mortality and particularly CV mortality in a cohort of 433 bronchiectasis patients enrolled in the TAYBRIDGE (Tayside Bronchiectasis Registry Integrating Datasets, Genomics, and Enrolment into Clinical Trials) bronchiectasis registry. This is in line with previous studies in COPD in which circulating desmosine concentrations were significantly higher in patients with a prior history of CV disease and were significantly associated with mortality [32]. The study also found that the combination of serum desmosine and BSI significantly improved the prediction over the BSI alone, especially among individuals classified as having severe disease.

In a retrospective analysis of a prospective study by Méndez *et al*. [30] including 250 bronchiectasis patients across two tertiary care hospitals in Spain, risk factors for CV events during and following pulmonary exacerbations were investigated. Over a median follow-up of 35 months, 29.6% experienced at least one CV event, and 37.2% ultimately died. Semi-competing risk analysis demonstrated that age, arterial hypertension, COPD and potentially severe exacerbations significantly increased the likelihood of CV events, as well as bronchiectasis patients and CV events showing a higher mortality rate.

### Quantitative evaluation of the CV risk in patients with bronchiectasis

**FIGURE 2:**
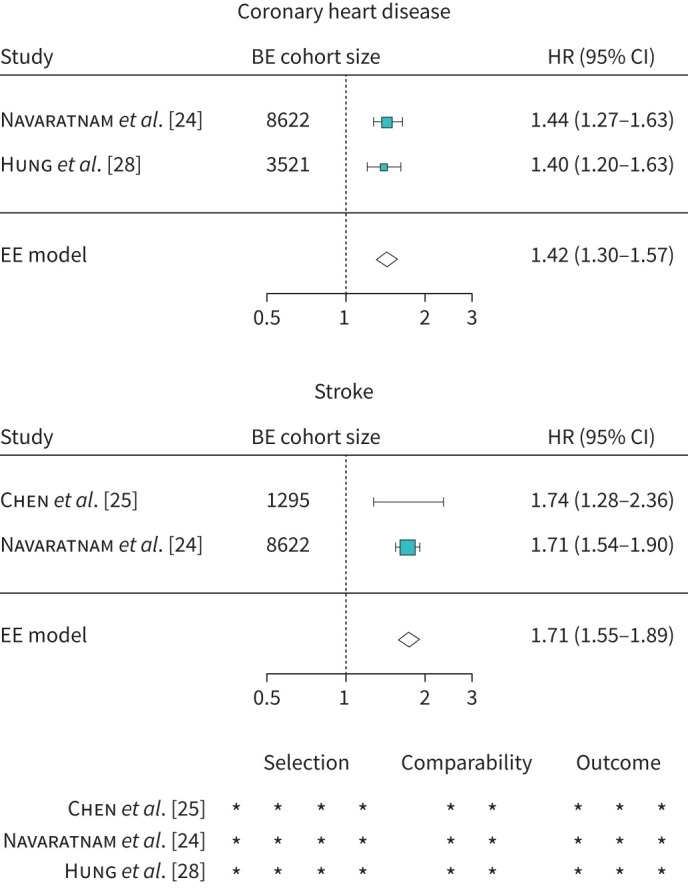
Pooled estimate of excess risk for coronary heart disease or stroke in patients with bronchiectasis (BE) and a quality assessment of the studies. The quality assessment was performed using the Newcastle-Ottawa Scale, with a maximum of four stars for group selection, two stars for group comparability and three stars for outcome ascertainment. HR: hazard ratio; CI: confidence interval.

Figure 2 shows the pooled estimates of the association between bronchiectasis and CHD or stroke obtained from three studies. The pooled HR were 1.42 (95% CI 1.30–1.57) for CHD and 1.71 (95% CI 1.55–1.89) for stroke.

### Quality assessment of the included studies

The quality of the included studies was generally high. Patients included were selected from national electronic databases or specialised tertiary hospitals, thus ensuring they were representative of the bronchiectasis population. The main critical point was the absence of a comparison group in five of the nine studies included in this systematic review [21, 26, 27, 29, 30], which prevented us from including them in the quantitative assessment of the excess CV risk in this population. Among the remaining four studies, the first one published in 2014 by Navaratnam *et al*. [31] was excluded from the meta-analysis because data on a larger cohort selected from the same primary care database were published in 2017 [24]. After the exclusion of this study, the three studies were all considered in the quantitative evaluation and received the maximum score in every domain of the Newcastle-Ottawa Scale, indicating that the pooled estimates of excess risk for CV events in the bronchiectasis population were derived from high-quality studies.

## Discussion

We present here the first systematic evaluation of CV events in individuals with bronchiectasis. Our study found high incidence of various CV events with pooled risk being 42% higher for CHD and 71% higher for stroke in bronchiectasis patients.

CV events have been previously reported to affect disease progression and clinical outcomes in several chronic respiratory diseases, with the majority of data derived from patients with COPD [33]. A landmark meta-analysis by Chen *et al.* [33] reported a 2–5-fold higher risk of ischaemic heart disease, arrhythmias and large artery disease in patients with COPD compared to controls. Pathophysiological mechanisms between COPD and CV disease might include shared risk factors (*e.g.* smoking), respiratory factors (lung hyperinflation and chronic hypoxemia) and systemic inflammation [34]. On the other hand, the coexistence of bronchiectasis and CV diseases has been addressed to date by a small number of studies with only fragmentary data on the prevalence and incidence of CV events. However, this association is biologically plausible and can be attributed to several underlying mechanisms. Many studies showed that local and systemic inflammation are increased in bronchiectasis patients [21]. Low-grade inflammation has been demonstrated to contribute to the development and progression of atherosclerosis, from endothelial dysfunction and formation of mature atherosclerotic plaques to plaque rupture and thrombosis [35]. Consistent with this, in a recent retrospective study Williams *et al.* [36] described high prevalence of coronary artery calcification in bronchiectasis patients, with more extensive coronary artery calcification in the group with severe bronchiectasis. Several studies showed a relationship between inflammation and vascular dysfunction in this population [35]. Inflammation plays an important role in stiffening of large arteries, endothelial dysfunction and vascular calcification, thus explaining the increased vascular risk in several inflammatory disorders. In 2012 Gale *et al.* [13] demonstrated increased pulse wave velocity as a validated technique for aortic stiffness in bronchiectasis patients. These findings were confirmed by other studies, which observed a significant correlation between carotid and femoral pulse wave velocity with disease severity and frequency of pulmonary exacerbations [11, 37, 38].

Pulmonary exacerbations may also play a role in this field and lead to a transient increase in the risk of CV events [39–42]. Again, most of data come from COPD research; in a recently published meta-analysis, for example, Müllerová *et al.* [43] found an increased risk of coronary syndrome or stroke within a relatively short period of time following a COPD exacerbation.

Extending the rationale to bronchiectasis patients, it is to be noted that most of the studies considered in this analysis involved patients during and after a bronchial exacerbation.

This study has both strengths and limitations. To our knowledge this is the first systematic evaluation of an increased risk of CV events in bronchiectasis patients both in terms of a qualitative review and quantification of the risk. The limited number of the studies included in the quantitative analysis is a major limitation for this study. It prevented us from stratifying participants based on clinical characteristics such as frequent exacerbations or disease severity and did not allow us to evaluate possible sources of heterogeneity across different studies conducted in various settings and countries.

### Conclusions

The coexistence between bronchiectasis and the risk of CV events underlines the importance of CV screening in this population. In terms of clinical implications, interventions aimed at increasing awareness of this association should be implemented in order to develop preventive strategies. Future research may investigate the mechanisms underlying the excess CV disease in patients with bronchiectasis. Large cohort studies are needed to provide accurate and precise estimates of the occurrence of CV events in this population. Although CV events are influenced by low-grade chronic inflammation and oxidative stress, further translational studies are needed to better clarify the interaction between respiratory and CV health.
